# Decreasing Blood Culture Contaminants in a Pediatric Emergency Department: An Interrupted Time Series Analysis

**DOI:** 10.1097/pq9.0000000000000104

**Published:** 2018-09-19

**Authors:** Paul C. Mullan, Sara Scott, James M. Chamberlain, Jeanne Pettinichi, Katura Palacious, Anastasia Weber, Asha S. Payne, Gia M. Badolato, Kathleen Brown

**Affiliations:** From the * Department of Pediatrics, Division of Emergency Medicine, Eastern Virginia Medical School, Children’s Hospital of the King’s Daughters, Norfolk, VA; † Division of Emergency Medicine, Children’s National Health System, Washington, DC; ‡ Department of Pediatrics, Division of Emergency Medicine, The George Washington University School of Medicine, Washington, DC.

## Abstract

**Introduction:**

High peripheral blood culture contamination rates (BCCR) in the emergency department (ED) contribute to overuse and harm. This study describes 2 years of quality improvement (QI) interventions that aimed to decrease a high BCCR in a pediatric ED.

**Methods:**

The QI team created a Key Driver Diagram with multiple Plan-Do-Study-Act (PDSA) cycles. PDSA interventions included a venipuncture sterility checklist (PDSA1), phlebotomist feedback system (PDSA2), and physician ordering guidelines (PDSA3). The specific aim was to decrease the BCCR by 50% within 24 months. The secondary aim was to decrease the peripheral blood culture ordering rate (BCOR) by 10% within 24 months. The balancing measure was the proportion of pathogenic bacteremia cases at ED return visits before and after PDSA3 implementation. A financial measure estimated the savings in charges between the observed and expected contaminants in PDSA3. An interrupted time series design applied statistical process control methodology to detect special cause variations.

**Results:**

The BCCR in the baseline, PDSA1, PDSA2, and PDSA3 periods were 3.02%, 2.30%, 1.58%, and 1.17%, respectively. The BCOR in the baseline, PDSA1, PDSA2, and PDSA3 periods was 4.80%, 4.26%, 3.82%, and 3.49%, respectively. Special cause variations occurred after PDSA cycle implementations for both BCCR and BCOR. There was no change in the balancing measure. The interventions were associated with an annual prevention of 95 contaminants and savings of $300,070.

**Conclusions:**

Interventions that focused on improving venipuncture technique and limiting unnecessary blood cultures were associated with fewer contaminants and the achievement of the QI team’s project aims.

## Abstract 2

Supplemental Digital Content is available in the text.

## INTRODUCTION

Blood cultures are commonly ordered in the emergency department (ED) setting to evaluate for bacteremia. In 2014, the National Hospital Ambulatory Medical Care Survey estimated that ED clinicians had obtained over 840,000 blood cultures on children in EDs in the United States.^1^ Contamination of blood cultures contribute to unnecessary return visits, diagnostic studies, and expenses.^2,3^ Depending on the age and condition of the patient, a contaminated blood culture may result in repeated blood culture, lumbar puncture, and/or hospital admission.

Although the national benchmark for blood culture contamination rates (BCCR) is 2–3%, many EDs have reported rates as high as 11%.^4–8^ Multiple studies in the ED have tested interventions to decrease blood culture contaminants, achieving contamination rates as low as 1.4%.^7,9–13^

In 2015, a group of ED nurses approached ED leadership at the Children’s National Medical Center with their concerns regarding patient harm and costs related to the high number of peripheral blood culture contaminants in their patients. The BCCR for the prior 12-month period, July 2014 to June 2015, was 3.02%, similar to a previously published BCCR of 2.9% from this institution between 1994 and 1996.^14^ ED leadership assembled a multi-disciplinary team of nurses and physicians to address this problem using quality improvement (QI) principles and the Model for Improvement framework.^15^ The team’s global aim was to reduce the number of peripheral blood culture contaminants. The specific aim was to decrease the BCCR by 50% within 24 months of project implementation. The team hypothesized that 2 key drivers would achieve their project’s aims: increasing venipuncture sterility and decreasing the number of blood cultures ordered.

## METHODS

### Setting

This prospective, single-center, interrupted time-series design QI project occurred in an urban, academic pediatric ED that sees approximately 90,000 patients per year. The ED is a level-one trauma center that is part of a free-standing children’s hospital with 320 inpatient beds. A trainee (eg, pediatric resident or emergency medicine resident) and an attending physician see the majority of ED patients. Registered nurses and ED patient care technicians perform all venipunctures. Henceforth, the term “phlebotomist” describes the ED member drawing the blood culture sample. The processing of blood culture samples was previously described.^14^ The study received exempt status from the institutional review board.

### Interventions

**Fig. 1.**
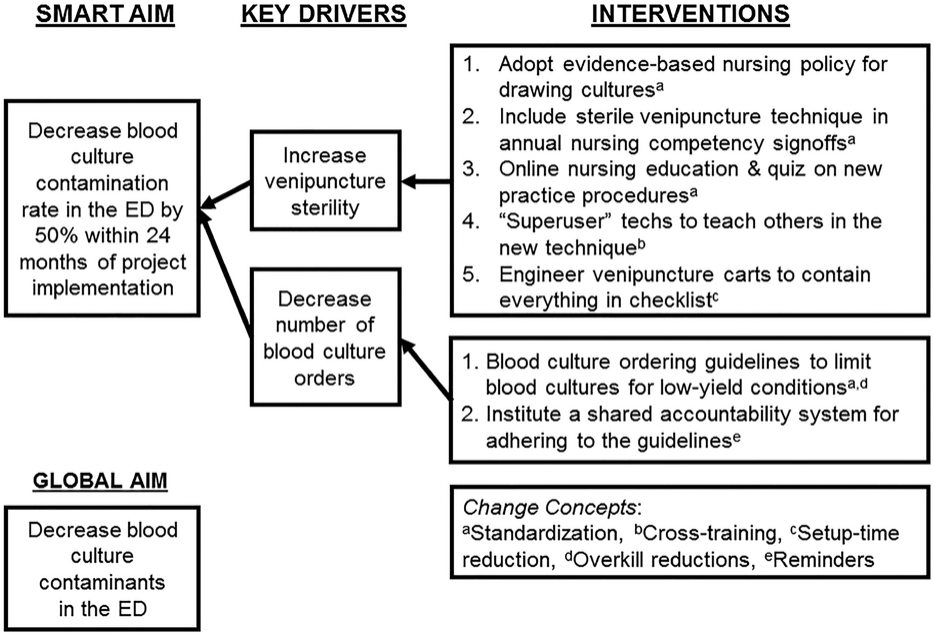
Key driver diagram.

The QI team consisted of 4 ED nurses and 4 ED physicians. Two of the QI team nurses were educational leaders, and QI team physicians included the medical director and the chief of the division. Other content experts included the chief of microbiology, ED technicians, pediatric emergency medicine fellows, and an ED statistician. The team built a key driver diagram (Fig. 1) to display the global aim, specific aim, and key drivers. Three Plan-Do-Study-Act (PDSA) cycles occurred during this project.

#### PDSA #1: Venipuncture Checklist

**Fig. 2.**
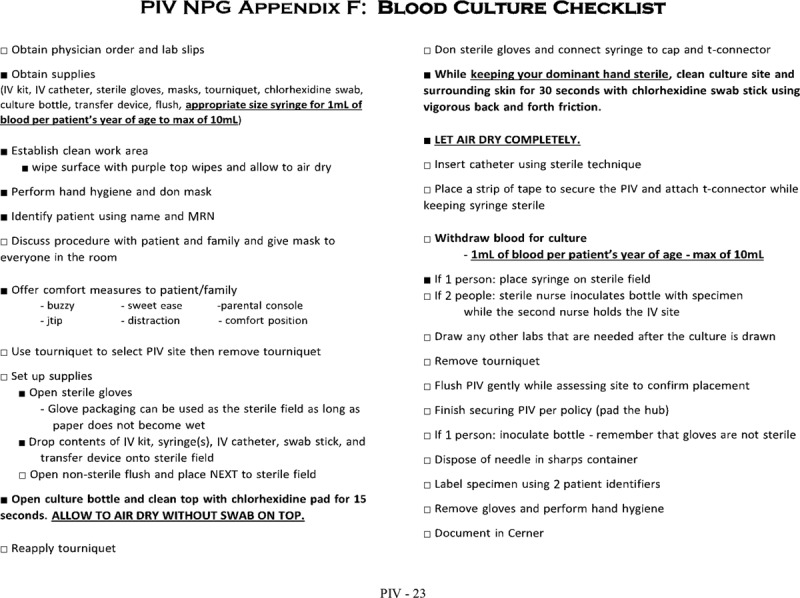
ED peripheral blood culture checklist. IV, intravenous; MRN, medical record number; NPG, nursing practice guideline; PIV, peripheral intravenous line.

For PDSA1, the QI team addressed the key driver of improving venipuncture sterility. The team reviewed the hospital’s phlebotomy policy and published interventions that had decreased BCCRs in similar settings.^7,9–13^ The QI team nurses discussed the feasibility of implementing evidence-based new practices phlebotomists at change-of-shift team huddles and in individual conversations. The QI team reviewed the feedback from this group and amended the hospital phlebotomy policy with specific instructions for drawing peripheral blood cultures (**Supplemental Digital Content 1**, available at http://links.lww.com/PQ9/A36). The primary additions to the policy included improving hand hygiene technique, and requiring phlebotomists to wear face masks, don sterile gloves, and scrub the culture site for 30 seconds before phlebotomy. A 30-item checklist documented covered all items from the policy. This checklist was affixed to the side of mobile phlebotomy carts for visibility at the point of care (Fig. 2).

Next, the QI team nurses changed the setup of the phlebotomy cart to have all supplies required for checklist completion in proximity to each other in 1 drawer. To reinforce knowledge and practice with the checklist, all ED nurses and technicians completed an online module with a knowledge assessment test, performed the checklist on a simulation model, and were required to repeat the simulated performance of the checklist as part of annual competency reviews. In addition, the QI team designated several phlebotomists as “superusers” who would coach other phlebotomists at the bedside on the new venipuncture process. The alterations in PDSA1 focused on key change concepts including standardization (ie, new policy), cross-training (superusers), and setup time reduction (ie, phlebotomy cart redesign). The start of PDSA cycle 1 (7/1/2015) was the announcement of the specific aim as an ED-wide quality goal by e-mail and staff huddles. This announcement coincided with the start of the period of checklist design, online training, and simulation training for the ED phlebotomists. Of the 150 ED phlebotomists, 98.7% completed the online module, and 94.7% completed the simulation model training.

#### PDSA #2: Phlebotomist Feedback

The start of PDSA2 (12/1/2015) initiated a feedback process to ED phlebotomists for contaminant blood cultures. Starting in PDSA2, an ED data analyst e-mailed the QI team a Microsoft Excel (v2013; Seattle, Oregon) document that listed all positive (pathogen or contaminant) and negative (no growth after 5 days) peripheral blood culture results from the prior week. If a positive culture was a contaminant, the nurses on the QI team reviewed the case with the patient’s phlebotomist, in person, to obtain feedback on barriers to the performance of the checklist and to review all of the items on the checklist.^15^

#### PDSA #3: Ordering Guideline

**Fig. 3.**
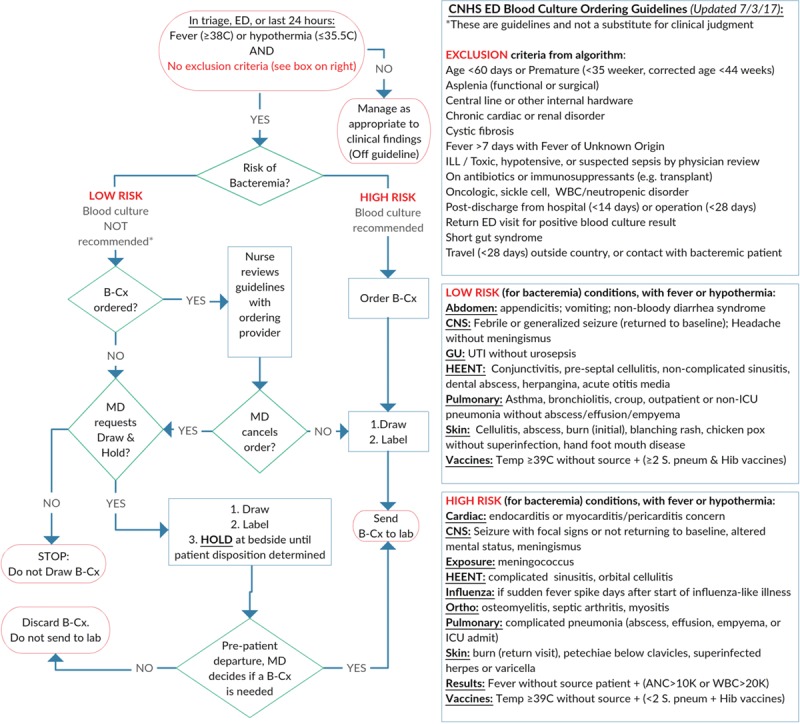
Blood culture ordering guideline. ICU, intensive care unit; WBC, white blood cell.

The start of PDSA3 cycle (7/1/2016) began when the QI team created and implemented the intervention of blood culture ordering guidelines. This guideline addressed the key driver of decreasing the number of blood culture orders (Fig. 3). To create this guideline, the QI team reviewed the primary diagnostic codes from the electronic health record of the 4,336 blood cultures from the 1-year baseline period. The QI team sorted the list alphabetically by the International Classification of Disease codes and assigned 2 ED physicians to each of the infection-related conditions with at least 5 occurrences during the baseline period, with at least 1 of the 2 physicians being a physician on the QI team. For each condition, the physicians assessed peer-reviewed papers, content expert analyses (eg uptodate.com), and evidence-based guidelines. The 2 physicians then reached consensus on categorizing the conditions into 1 of 3 categories: (1) low-risk (patient with either a very low risk for bacteremia and/or a low chance that the blood culture results would lead to a change in clinical management); (2) high-risk (blood culture indicated due to either a high risk of bacteremia and/or high chance that the blood culture results would lead to a change in clinical management); or (3) excluded from the guideline because blood cultures were indicated based on other guidelines (eg, leukemia patient with fever).

The QI team incorporated all the conditions and guideline recommendations into a process map (Fig. 3). In the ED, physicians first determined if the guideline applied to their patient based on whether the patient had an abnormal temperature (≥ 38°C or ≤ 35.5°C) and had no exclusion criteria. If the patient was not on the guideline, an alternate ED guideline or appropriate clinical judgment was applied. If the patient met criteria for the guideline and had a high-risk condition, the guideline recommended that the physician order a blood culture, after which the phlebotomist would draw the blood and send it to the laboratory.

If the patient met criteria for the guideline and had a low-risk condition, the physician had the option of requesting that the ED phlebotomist draw, label, and hold a blood culture sample at the bedside for up to 4 hours.^16^ The blood culture could later be ordered and sent to the laboratory if warranted by patient condition or disposition. If the physician ordered a blood culture on a patient with a low-risk condition, the ED phlebotomist was empowered, during their simulation training, to huddle with the ordering physician and review the blood culture ordering guideline as a part of a real-time shared accountability process. The ED physician would make the final determination to cancel the order, cancel the order but verbally request to draw and hold a blood culture at the bedside, or send the blood culture to the laboratory.

All contents of the guideline were distributed to all ED physicians by e-mail and reviewed at 2 consecutive monthly division meetings. The QI team incorporated ED physician feedback into the guideline before implementation. All ED physicians then completed a mandatory online quiz to educate themselves on the guideline content. Of the 81 ED physicians, 71 (88%) completed the online quiz, with an average of 92% of questions answered correctly. The guideline was also available in the ED on laminated posters on walls near the computer order entry area and phlebotomy carts.

### Inclusion and Exclusion Criteria

The QI team reviewed all data reports from the electronic health record of all ordered peripheral blood cultures for the baseline period and each week during the intervention period. Cultures were excluded from analysis if the patient had a central line, ventriculoperitoneal shunt, oncologic condition, neutropenia, or a history of a transplant. Contaminant and pathogen classifications were determined prospectively from prior studies (**Supplemental Digital Content 2**, available at http://links.lww.com/PQ9/A37).^5,9,14^

### Measures and Analysis

This interrupted time series study used statistical process control (SPC) charts to determine special cause variation.^17^ The project’s specific aim was to decrease the BCCR by 50% within 24 months. A 50% relative decrease was the median relative decrease from 6 published QI projects that focused on reducing peripheral blood culture contaminants in pediatric patients.^7,9–13^ An SPC T chart displayed the time in days between each contaminant event. Standard T chart special cause rules included 1 point outside of the control limits (3 SD) from center line, ≥ 4 of 5 points > 1 SD from center line on the same side, ≥ 2 of 3 points > 2 SD from center line on the same side, ≥ 8 points on the same side of center line, and ≥ 6 consecutive points increasing or decreasing.^17^

The secondary aim was a relative decrease in the blood culture ordering rate (BCOR) among all ED patients by 10% over 24 months. The SPC Proportion (P) chart special cause rule was 1 point outside of the control limits (> 3 SD) from the center line. Because asking ED physicians to decrease their BCOR might lead to potential harm by missing bacteremia patients, the proportion of missed bacteremia patients with an ED return visit less than 48 hours after discharge was the project’s balancing measure, with a goal of not significantly increasing this proportion. The numerator was the number of patients with pathogenic blood cultures on a second ED visit within 48 hours, and the denominator was the number of patients with a blood culture obtained on ED visit 2 with no blood culture obtained on ED visit 1. The proportions for this balancing measure were compared between 2 time periods: before (baseline, PDSA1, and PDSA2) and after PDSA3 implementation.

The financial measure estimated the savings in charges based on differences between the expected and actual number of contaminants seen in the PDSA3 cycle. We calculated the expected number of PDSA3 contaminant cultures by multiplying the annual number of patients during the PDSA3 cycle by the BCCR and BCOR of the baseline period. If we assume the adjusted inflation rates to 2017 dollars, the expected charges were $3,166 per contaminant.^7,18^

## RESULTS

**Fig. 4.**
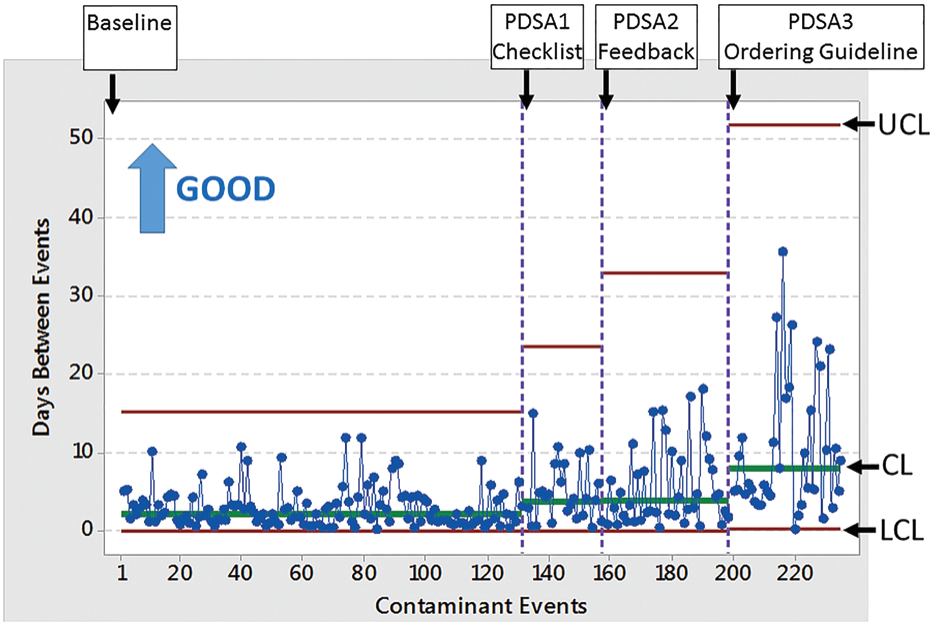
SPC T chart of the time in days between peripheral blood culture contaminant results in the ED. ANC, absolute neutrophil count; B-Cx, blood culture; CL, center line; CNHS, Children’s National Health System; CNS, central nervous system; GU, genito-urinary; HEENT, head, eyes, ears, nose, throat; Hib, *H. influenzae* b; LCL, lower control limits; MD, physician; Ortho, orthopedic; UCL, upper control limits.

The BCCR decreased from 3.02% (baseline) to 2.30% during PDSA1 (venipuncture checklist). During PDSA2 (phlebotomist feedback), the BCCR decreased to 1.58%. During PDSA3 (ordering guideline), the BCCR decreased to 1.17%. The project’s specific aim was achieved, with a 61% relative decrease in the BCCR. The center lines on the T chart improved from a baseline frequency of 1 contaminant every 2.2 days to a frequency during PDSA1, PDSA2, and PDSA3 cycles of 1 every 3.7 days, 4.1 days, and 7.8 days, respectively (Fig. 4). Special cause variation occurred in PDSA1 (4 of 5 points more than 1 SD from the prior center line) and PDSA3 (at least 8 points above the prior center line and 1 point above upper control limits). Regarding the stability of the baseline process, only 2 special cause variations occurred during 131 events: 1 instance of 4 of 5 points more than 1 SD on the same side below the center line, and 1 instance of ≥ 8 points on the same side below the center line. On chart review, we could not identify any reasons for these special causes.

**Fig. 5.**
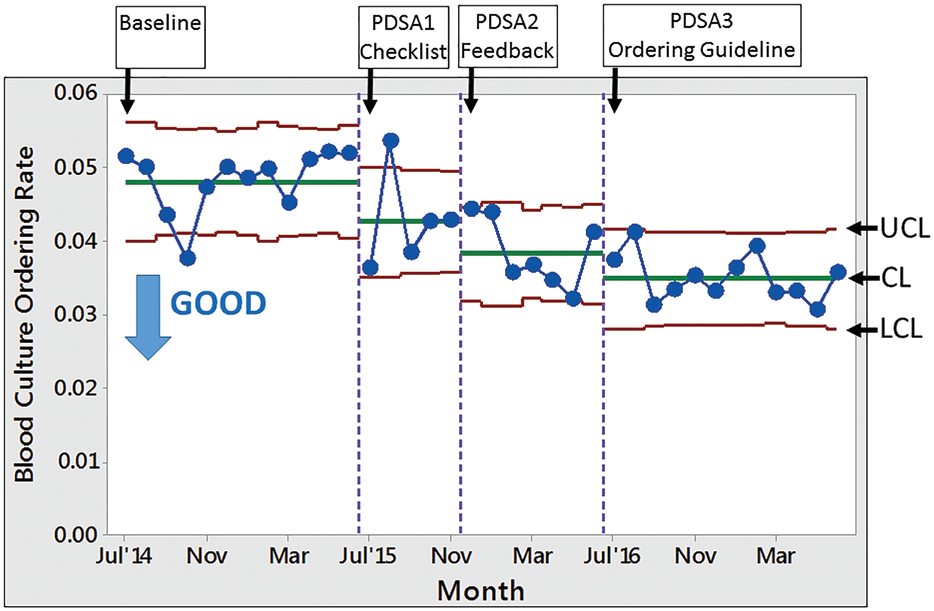
SPC P chart of the proportion of patient in the ED who had a peripheral blood culture ordered during the implementation. CL, center line; LCL, lower control limits; UCL, upper control limits.

For the secondary aim, the BCOR decreased from 4.80% (baseline) to 4.26%, 3.82%, and 3.49% during PDSA1, PDSA2, and PDSA3, respectively. The secondary aim was achieved with a 27% relative decrease (Fig. 5). Special cause variations occurred in all PDSA cycles, each with a point outside the lower control limits. The BCOR was stable during the baseline period at 4.8%.

For the balancing measure, there were 4 (3.6%) pathogenic bacteremia cases out of 111 ED return visits before PDSA3 implementation. After PDSA3 implementation, there was 1 pathogenic bacteremia case (2.3%) out of 44 ED return visits (difference of 1.3%, 95% confidence interval −4.3%, 6.9%).

For the financial measure, there were 88,454 patients in PDSA3. The expected number of contaminants was 131, and the actual number was 36; this reduction of 95 contaminants in PDSA3 amounted to annual savings in charges of $300,070.

## DISCUSSION

BCCRs in the ED setting have historically been higher than benchmark rates, contributing to unnecessary return visits, diagnostic tests, and expenses.^3,6–9,14^ Risk factors in the ED contributing to higher contamination rates include time pressures, phlebotomy during resuscitations, and high staff turnover.^8^ Using the Model For Improvement, a multi-disciplinary approach, and multiple PDSA cycles, the QI team achieved their specific aim of significantly reducing BCCRs. Other pediatric ED QI initiatives have demonstrated decreased contamination rates by improving antisepsis technique,^7^ educating phlebotomists on sterile technique,^7,13^ providing feedback to phlebotomists on contaminants,^7,13^ designating select phlebotomists to draw cultures,^13^ and requiring a separate venipuncture site for blood cultures.^9,11,12^ After considering the feasibility of these interventions, the QI team adopted all these interventions except using select phlebotomists and using a separate venipuncture site, to maximize staffing capacities and limit patient pain, respectively. Additional interventions not previously described in the pediatric ED setting were annual nursing competency assessments, super-user phlebotomists for continuous staff refreshers, and reconfiguration of the phlebotomy cart. These additional interventions might have contributed to this project’s low BCCR of 1.17%, the lowest published BCCR from a pediatric ED setting. The duration of the project’s intervention phase (24 months) was longer than those described in other studies (median, 7.5 months; range, 3–12 months), strengthening the degree of belief that the gains will be sustainable.^7,9–13^

The QI team achieved its secondary aim with a 27% relative decrease in the BCOR. Two similar QI studies have reported relative decreases in BCOR of 4% (Murillo et al.^10^, 11.8% to 11.4%, not significant) and 16% (Hall et al.^7^, 7.2 to 6.1%, *P* < 0.0001). Only the latter study commented on this decrease, attributing it to a potential Hawthorne effect. The Hawthorne effect might have explained the initial decreases in BCOR during the project’s PDSA1 and PDSA2 cycles. A second potential effect during PDSA1 and PDSA2 was that several ED physicians were concurrently designing the ordering guideline and obtaining feedback on the design of the ordering guidelines from many of the ED physicians. The QI team was unaware of any similar blood culture ordering guidelines in either the pediatric or adult ED literature. This guideline and the training of ED phlebotomists to promote shared accountability with physicians might account for the greater relative decrease in BCOR than was seen in the other pediatric studies. Importantly, the decrease in BCOR in this study was not associated with a concomitant increase in missed bacteremia cases, a balancing measure that was not included in prior studies.

The blood culture ordering guideline and implementation process focused on key change concepts including standardization (ie, ordering guideline), reducing over-testing (ie, recommending no blood culture for low-risk conditions), and reminders (ie, guideline posters, annual quizzes, and shared accountability).^15^ The design of the guideline attempted to address well-described barriers to physician adherence to practice guidelines related to lack of awareness of a guideline, lack of agreement with guidelines, lack of outcome expectancy, lack of self-efficacy, and environmental factors.^19^

In addition to demonstrating fewer contaminants, the QI team demonstrated a financial impact of over $300,000 per year in estimated savings in charges, similar to the Hall et al.^7^ reported annual savings of $250,000. While charges are not equal to costs, charges are a surrogate measure of financial impact. These charges accounted for savings from fewer contaminant cultures and did not include additional savings realized by lower BCORs for all ED patients. These calculations did not measure other economic and psychosocial costs associated with fewer false-positive laboratory tests such as missed work days, pain associated with procedures, and additional stress on patients, caregivers, and ED staff. As health care evolves into value-based care models, decreasing preventable events such as blood culture contaminants might become a financial incentive for EDs.

One limitation of this study was its single-center design, limiting the potential generalizability to other ED settings. However, this project and others published on the topic of blood culture contaminants have successfully addressed the problem with bundled interventions containing similar components. Although this study was not designed to determine the relative strength of each component, a bundled approach is probably the most feasible and practical. It is possible that missed bacteremia cases went to other EDs in the region, although this study’s ED would have likely received such patients as transfers, given that they are the region’s primary children’s hospital.

By implementing multiple interventions that focused on the key drivers of improving venipuncture sterility and decreasing the number of blood culture orders, our QI team achieved a significant reduction in the number of peripheral blood culture contaminants.

## DISCLOSURE

The authors have no financial interest to declare in relation to the content of this article.

## Supplementary Material

SUPPLEMENTARY MATERIAL
