# Supplementary material for: Decreasing Blood Culture Contaminants in a Pediatric Emergency Department: An Interrupted Time Series Analysis
Source: Pediatr Qual Saf. 2018 Sep 19;3(5):e104. doi: 10.1097/pq9.0000000000000104 (PMC6221596; doi:10.1097/pq9.0000000000000104)
Supplement: SUPPLEMENTARY MATERIAL [file pqs-3-e104-s001.docx]

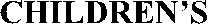

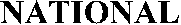

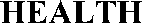

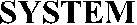
Appendix E


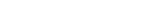

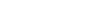

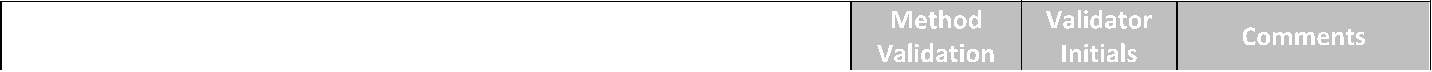

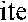

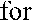

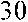

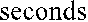

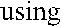

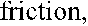

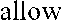

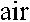

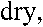

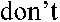

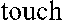

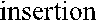

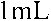

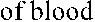

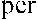

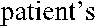

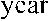

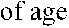


|  |  | | |
| --- | --- | --- | --- |
| Identify patient populations needing blood cultures. | VF D |  |  |
| Explain rationale for not drawing a blood culture from an already established IV site. | VF D |  |  |
| Explain rationale of why blood cultures should be collected before any other blood samples are collected from the same site. | VF D |  |  |
| Verbalize the most common source of blood culture contamination (bacteria from the skin) and methods of decreasing contamination (scrub  site, wear sterile gloves, use adequate volume of blood). | VF D |  |  |
| Describe correct amount of blood to be drawn for a blood culture. to a max of 10mL | VF D |  |  |
| Identify and collect appropriate supplies for procedure. Comfort measure (jtip, buzzy, sweet ease) ChloraPrep swab stick and pad  Blood culture bottle Tourniquet Syringe(s)  Butterfly needle/IV catheter IV start kit  Sterile gloves  Gauze pad and bandaid (if venipuncture) Saline flush (if inserting PIV) | D RD |  |  |
| Demonstrate proper hand hygiene and standard precautions. | RD |  |  |
| Identify the patient using two identifiers per hospital policy. | RD |  |  |
| Discuss the procedure with patient and family. Provide opportunity for patient and family to ask questions and have concerns addressed prior to beginning procedure. Prepare patient using developmentally appropriate methods and resources (e.g. Child Life Specialists). | RD |  |  |
| Discuss available options for Comfort Measures for procedure with patient and family. Consider both pharmacologic and non-pharmacologic adjuncts (see Practice Guideline). | RD |  |  |
| Assess patient for all potential venipuncture sites, applying tourniquet briefly as necessary. Offer patient choices when appropriate, using clinical judgment. Utilize transilluminator or other hospital-approved vein finding device when needed. | RD |  |  |
| Position patient appropriately for procedure, utilizing Bear Hugs when possible. Ensure selected extremity is stabilized and able to be controlled safely. | RD |  |  |
| Establish clean work area. | RD |  |  |
| Demonstrate proper hand hygiene and don sterile gloves. | RD |  |  |
| Set up supplies while maintaining sterility. | RD |  |  |
| Clean top of culture bottles with CHG and allows to air dry completely. | RD |  |  |
| Cleanse skin with chlorhexadine gluconate (CHG) for 30 seconds unless contraindicated (see Skin Care Nursing Practice Guideline). Allow to dry for 30 seconds. Do not touch skin after it is cleaned, even with a gloved finger. | RD |  |  |

| **NAME:** | **DEPT/UNIT:** |
| --- | --- |
| **EMPLOYEE** **ID:** | **DATE** **COMPLETE:** |

COMPETENCY VALIDATION CHECKLIST: Peripheral Blood Culture Competency

COMPETENCY STATEMENT: Successfully demonstrate proper procedure for obtaining peripheral blood culture. Validation Key: VF = Verbal Feedback, OB = Observation, D = Discussion, RD = Return Demonstration

PIV - 21


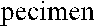

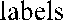

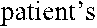

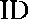

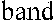

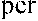

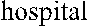

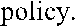


| Reapply tourniquet proximal to site. Leave in place for the shortest time necessary to complete procedure, release and reapply if needed for longer than 2 minutes or if any circulation compromise is noted. | RD |  |  |
| --- | --- | --- | --- |
| Perform venipuncture or peripheral IV insertion while maintaining sterility. | RD |  |  |
| Draw correct specimen volume according to guidelines above. | RD |  |  |
| Directly inoculate the blood culture bottles using angel wings. | RD |  |  |
| Dispose of needle in the sharps container. | RD |  |  |
| Remove gloves and perform hand hygiene. | RD |  |  |
| Label all specimens at the bedside, matching two patient identifiers on | RD |  |  |
| Document procedure in the medical record. | RD |  |  |

| **Preceptor** **Name** **(Please** **Print)** | **Initials** | **Date** |
| --- | --- | --- |
|  |  |  |
|  |  |  |

*Updated* *8/2015*

PIV - 22
