# Supplementary material for: Decreasing Blood Culture Contaminants in a Pediatric Emergency Department: An Interrupted Time Series Analysis
Source: Pediatr Qual Saf. 2018 Sep 19;3(5):e104. doi: 10.1097/pq9.0000000000000104 (PMC6221596; doi:10.1097/pq9.0000000000000104)
Supplement: SUPPLEMENTARY MATERIAL [file pqs-3-e104-s002.docx]

Supplemental Digital Content – Table 1. Criteria for classification of positive peripheral blood cultures as pathogenic or contaminant bacteria.

| Pathogen | Contaminant |
| --- | --- |
| *Acinetobacter* (not *lwoffii*) | *Acinetobacter lwoffii* (oral flora) |
| *Bordetella holmesii* | *Bacillus species* (not *anthracis*) |
| *Candida albicans* | *Bacteroides fragilis* |
| *Citrobacter freundii* | *Clostridum perfringens* |
| *Clostridium non-perfringens* | *Corynebacterium species* |
| *Escherichia coli* | *Cryptococcus* |
| *Enterobacter cloacae* | Gram positive bacilli (diphtheroid like) |
| *Enterococcus faecalis/faecium* | Incomplete growth |
| *Haemophilus influenzae* | *Microbacterium* |
| *Klebsiella pneumoniae* | *Micrococcus* |
| *Moraxella catarrhalis* | More than one organism grew from sample |
| *Neisseria meningitidis* | *Neisseria* (not *meningitidis)* |
| *Pseudomonas aeruginoas* | *Peptostreptococcus* |
| *Salmonella species* | *Propionobacterium acnes* |
| *Serratia marcescens* | *Roseomonas* |
| *Staphylococcus aureus* | *Staphylococcus capitus* |
| *Staphylococcus saprophyticus* | *Staphylococcus epidermidis* |
| *Streptococcus agalactiae* | *Staphylococcus haemolyticus* |
| *Streptococcus pneumoniae* | *Staphylococcus hominis* |
| *Streptococcus pyogenes* | *Staphylococcus* species coagulase negative |
|  | *Streptococcus* species (not *enterococcus*) |
|  | *Streptococcus viridans* |
